# No straight lines – young women’s perceptions of their mental health and wellbeing during and after pregnancy: a systematic review and meta-ethnography

**DOI:** 10.1186/s12905-019-0848-5

**Published:** 2019-12-05

**Authors:** Grace Lucas, Ellinor K. Olander, Susan Ayers, Debra Salmon

**Affiliations:** Centre for Maternal and Child Health Research, School of Health Sciences, City, University of London, Northampton Square, London, EC1V 0HB UK

**Keywords:** Depression, Mental health, Meta-ethnography, Pregnancy in adolescence, Systematic review, Wellbeing, Teenage pregnancy, Young mothers

## Abstract

**Background:**

Young mothers face mental health challenges during and after pregnancy including increased rates of depression compared to older mothers. While the prevention of teenage pregnancy in countries such as the United States and the United Kingdom has been a focus for policy and research in recent decades, the need to understand young women’s own experiences has been highlighted. The aim of this meta-ethnography was to examine young women’s perceptions of their mental health and wellbeing during and after pregnancy to provide new understandings of those experiences.

**Methods:**

A systematic review and meta-ethnographic synthesis of qualitative research was conducted. Seven databases were systematically searched and forward and backward searching conducted. Papers were included if they were from Organisation for Economic Co-operation and Development countries and explored mental health and wellbeing experiences of young mothers (age under 20 in pregnancy; under 25 at time of research) as a primary research question – or where evidence about mental health and wellbeing from participants was foregrounded. Nineteen papers were identified and the Critical Appraisal Skills Programme checklist for qualitative research used to appraise the evidence. Following the seven-step process of meta-ethnography, key constructs were examined within each study and then translated into one another.

**Results:**

Seven translated themes were identified forming a new line of argument wherein mental health and wellbeing was analysed as relating to individual bodily experiences; tied into past and present relationships; underpinned by economic insecurity and entangled with feelings of societal surveillance. There were ‘no straight lines’ in young women’s experiences, which were more complex than dominant narratives around overcoming adversity suggest.

**Conclusions:**

The synthesis concludes that health and social care professionals need to reflect on the operation of power and stigma in young women’s lives and its impact on wellbeing. It adds to understanding of young women’s mental health and wellbeing during and after pregnancy as located in physical and structural factors rather than individual capacities alone.

## Background

Countries such as the United Kingdom and the United States have high teenage pregnancy rates compared to other countries [1, 2]. Although rates of teenage pregnancy have been falling in these countries [3, 4] there has been a continued focus on teenage or adolescent pregnancy (under the age of 20) [5] as a particular social and public health problem [6].

The mental health challenges faced by young women both during and after pregnancy have been highlighted in recent research [7, 8]. It has been reported that pregnant teenagers may be at an increased rate of depression compared to non-pregnant teens and adults [9, 10] with prenatal depression higher in young pregnant women today than in previous generations [11]. Postpartum depression among pregnant teenagers also has a prevalence that is up to double that observed in adult mothers [12].

Not all young women who are pregnant or parenting have mental health issues – and for some young women parenting is a positive and affirming experience – but problems may be particularly pervasive in high-risk groups such as those who lack support [13], for those young women who have experience of the care system [14] or those with prior psychological distress [15]. Furthermore, young women who have suffered abuse [16] or have been exposed to violence may also be at risk for increased depressive symptoms [17, 18]. Poverty is also recognised as a key contributing factor to young pregnant and parenting women’s mental health problems [9, 19].

Internationally, a range of policies and practice guidelines have been developed around preventing teenage pregnancy and helping to improve outcomes for young women and their children [20, 21]. In response to this framing of teenage pregnancy as problematic in some countries, the importance of hearing young women’s own voices has been emphasised [22]. To date, no reviews have been published synthesising qualitative literature on the emic perspectives of young mothers on their mental health and wellbeing. The aim of this synthesis was to address this gap by synthesising studies of young women’s mental health and wellbeing experiences during and after pregnancy. The rationale for choosing a meta-ethnographic approach is because – in line with its ethnographic origins – meta-ethnography seeks to stay close to the emic perspectives of participants [23]. Given that the views of young women during and after pregnancy have been reported to be underrepresented, this aspect of meta-ethnography provides a way to stay close to their experiences. However, at the same time, the focus of this integrative (as opposed to aggregative) methodology is concerned with creating new interpretation and understanding [24, 25], which is important in moving forward the field.

Whilst any research focused on young mothers’ perspectives might touch on their overall experiences, for the purposes of this synthesis, we focused specifically on perceptions of their mental health and wellbeing. Wellbeing was defined as including overlapping concepts of mental wellbeing (including, but not limited to, levels of stress, self-esteem, self-confidence), social wellbeing (including, but not limited to, levels of social connectedness, social cohesion) and physical wellbeing (including, but not limited to, nutrition, sleep, physical activity) [26]. The focus was also on common mental health problems as reported by young women participants, such as depression and anxiety and was driven by the question: what are young women’s perceptions of their mental health and wellbeing during and after pregnancy?

## Methods

### Search strategy and process

To develop the qualitative search, we used the PEO (Population, Exposure, Outcome) tool [27]. We refined search terms during a scoping process to search for qualitative studies on young women’s (P) experiences and perceptions (O) of their mental health and wellbeing (E) during and after pregnancy. The search was tested in Medline and then adapted for other databases. Each category included medical subject headings (MeSH) and keywords using trunctation (*) within title or abstract fields. The review was registered with Prospero (registration number CRD42018096641) and eMERGe reporting guidelines for meta-ethnographies were followed [28].

One researcher (GL) conducted a systematic search of seven electronic databases in July 2018: Medline, EMBASE, CINAHL, Web of Science, PsycINFO, Amed and Scopus. Boolean terms “OR” and “AND” were used to combine searches within and between categories respectively. The database searches were limited to the last 20 years (1998–2018). The rationale for this time period was that although rates of teenage pregnancy, in countries that have complete statistics, have been falling since the mid 1990’s [2] the last 20 years also coincides with teenage motherhood being framed as a social and public health problem in countries such as the USA and the UK [29] with policies developed to reduce teenage pregnancy rates [20]. The reference lists of all potentially relevant papers were examined (backward searching) and forward searching using the database Scopus was conducted to identify any additional potentially relevant articles where these papers had been cited.

### Selecting primary studies

Titles and abstracts from the searches were screened against the inclusion criteria by one researcher (GL) to exclude irrelevant papers. Ten per cent of titles/abstracts were also independently reviewed by another team member (EO) to confirm exclusion decisions. To be included in this review, studies had to either take mental health or wellbeing as a primary research question or report primary evidence from participants that focused on their perceptions of their mental health and wellbeing. This meant that studies which took a very general view of young women’s experiences of motherhood were not included in this particular synthesis to enable a focused response to the question. Indeed, given that a meta-ethnography should be mindful of context, it was decided to limit the search to research conducted in Organisation for Economic Co-operation and Development (OECD) countries only, due to the broad commonalities between these countries in trends around age of mothers at childbirth. The inclusion and exclusion criteria are outlined in Table 1.

**Table 1:** Inclusion and exclusion criteria

Inclusion criteria:	
• Studies exploring as a primary research question or objective mental health and wellbeing experiences of young mothers (age under 20 at time of pregnancy, and under 25 at the time of the research) in pregnancy and the post-natal period (up to one year after birth).	
• Studies where primary evidence from young mothers foregrounds their mental health and wellbeing experiences in pregnancy and the postnatal period (up to one year after birth).	
• Studies which employ qualitative methods (pure or mixed methods) of data collection and analysis	
• Studies from OECD countries.	
Exclusion criteria:	
• Studies with young mothers who were over the age of 20 at the time of pregnancy or over the age of 25 at data collection.	
• Studies where the views of young mothers (under the age of 20) are included but not separately identified or reported.	
• Studies with young women on pregnancy termination.	
• Studies which exclusively investigate the experience of pharmacological or service development interventions for mental health problems.	
• Studies without a primary focus on mental health and wellbeing in the research question or study aims or primary evidence from young mothers’ accounts.	
• Studies that discuss issues related to mental health and wellbeing (i.e. stigma or violence) but where no connection is explicitly referred to in study aims or in participants’ accounts.	
• Studies with quantitative methods of data collection and analysis.	
• Qualitative data with no verbatim quotes.	
• Reviews, opinion pieces/commentaries, theses and dissertations, non-peer reviewed journal articles, reports, conference abstracts.	
• Studies from non-OECD countries.	

Each full-text paper was reviewed independently by two team members followed by a decision to include or exclude. Where consensus could not be agreed, a third team member was asked to decide.

### Reading, data extraction and quality appraisal approach

Each of the included papers were read in full by GL and additionally read by another team member. GL was responsible for data extraction, and EO and DS reviewed the extraction tables.

Both the appraisal and exclusion of qualitative papers as part of a systematic reviews and metasynthesis is an area of debate [30, 31]. In common with other syntheses [32, 33] we took an inclusive approach and chose not to exclude any papers based on quality assessment. However, we used the CASP criteria for qualitative research as a means of assessing the papers [34]. Although the CASP for qualitative research is not a recognised tool for scoring, other metasyntheses have used a score of seven out of ten on the CASP checklist as a threshold for a ‘reasonable quality’ paper [35, 36]. With this knowledge, GL appraised all the papers and a second reviewer (EO) further assessed a sample of these. In the subsequent analysis, whilst all papers were included and contributed to the findings, it was ensured that each of the final themes (and sub-themes) was supported by at least two papers considered to be of ‘reasonable quality’.

We followed the process of the identification of first and second order constructs as outlined in a meta-ethnography by Malpass et al. [37] GL extracted all the first order constructs (young women’s views of their experiences taken from the included studies) and second order constructs (authors’ interpretations taken from the studies), which were relevant to the question of women’s perceptions of their mental health or wellbeing, into two separate columns on a table. The full paper was checked for relevant second order constructs, although most conceptual data came from the findings or discussion sections. This was repeated for each primary study until all the constructs from all the papers were in one document.

### Determining how the studies are related

In this ethnography, the comparison of studies centred on primary and secondary constructs concerning the meanings of mental health and wellbeing in young women’s lives in the primary study accounts. A third column was created on the table wherein GL began to make initial comments about possible commonalities or differences between the papers. This was the beginning of the process for understanding how the studies might relate to one another. As all the data was within the single document it was possible to move back and forward between the studies to determine some initial ideas about how the studies might relate.

### Process of translating studies

A second table was then created. GL conducted the initial translation by placing the first primary study into a row, and then creating column headings from each of its second order constructs (documented in the first table). In order to retain the internal structure of arguments within each paper [38], and keep close to the context of the primary study, a column was also added to make note of the study’s overarching narrative argument. Under each column heading (construct), text (taken from the paper – mostly second order data) was inserted into the cell to support and explain it. As a row was added for each subsequent paper, a process of constant comparison made it clear that column headings (constructs) from some studies could encompass others as constructs were similar – the language and constructs from some papers therefore came to represent the overall themes of the meta-ethnography. All the second order constructs were re-examined at the end of this process to ensure the rigour of this process.

### Synthesis process

As with the study by Malpass et al. [37], for each of the translated constructs we created a ‘summary definition’, drawing once again from the language used in the original studies. ‘Third order’ interpretations (themes) [38] were then developed, with the translated second order constructs forming the sub themes. These were then placed into a new conceptual map to illustrate a line of argument – defined as a new 'storyline' or encompassing explanation of the phenomenon [39]. To establish the trustworthiness of findings, the research team discussed and debated the third order constructs/themes; considering other possible translations but arriving at a consensus on the synthesis. As modelled by Dheensa et al. [32] quotations (first order constructs) for each of the themes within the synthesis help to draw back to the words of the participants from the original studies to ensure this emic perspective was not lost in constructing the higher-level synthesis.

## Results

### Outcome of study selection

**Fig. 1:**
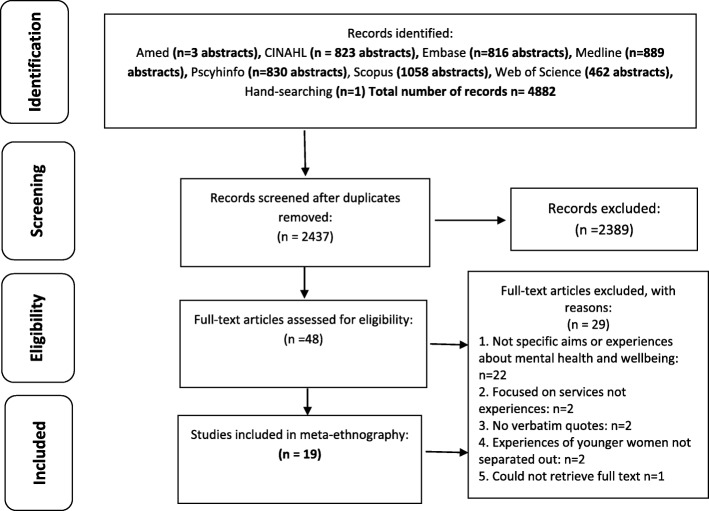
Outcome of study selection: PRISMA flow diagram

A total of 4882 records were retrieved, and 2455 duplicates removed. 2437 titles and abstracts were reviewed and 2389 were excluded. 48 papers were read in full and screened for inclusion – 29 of these were excluded, leaving 19 included papers [40–58]. The search process and outcome of study selection are outlined in Fig. 1: **–** Outcome of study selection: PRISMA flow diagram.

### The synthesis papers: characteristics

Nineteen papers detailing nineteen independent studies met the inclusion criteria (see Table 2). All studies were published in English between 1999 to 2017. Fourteen studies were based in the USA, four in the UK and one in Canada and all sampled English speaking participants only. All studies aimed to report the experiences of young women with specific focus in terms of mental health and its impact on various aspects of motherhood (*n* = 6) [40, 41, 45, 52, 55, 58], violence and abuse (*n* = 7) [42, 43, 46, 48, 49, 53, 54], socioeconomic experiences including housing and homelessness (*n* = 3) [47, 50, 51] and repeat pregnancies (*n* = 1) [57]. Two studies took a more general approach around the meanings of health or the role of support [44, 56]. In terms of the study objectives, three studies explicitly stated that informing prevention of teenage pregnancy was an objective driving the research [46, 54, 57], two studies foregrounded a mental health treatment/therapy objective [45, 55] and three studies mentioned a specific policy focus [43, 50, 53]. Other objectives included helping to improve outcomes or providing better services and support for young mothers [42–44, 47, 48, 50–53, 56–58] and understanding and prioritising young women’s voices [40, 41, 43, 44, 46, 49, 52–58]. Two studies took a feminist methodology [43, 58], one took a critical psychological perspective [50], one drew on relational-cultural theory [48], two studies drew from identity and role developmental theories [40, 56] and one worked within a psychotherapeutic model [45]. Six studies were explicitly framed by a positive, anti-deficit view of teenage motherhood [40, 43, 44, 49, 54, 56]. More generally, four studies included phenomenological approaches [41, 47, 54, 55], and three ethnographic methods [40, 44, 56], one drew on grounded theory [49]. Other studies did not report specific theoretical frameworks.

**Table 2:** Study characteristics of papers to be synthesised

Source paper and country	-Young women sample N -Age range -Pregnant/parenting stage (where provided)	-Ethnicity (as defined by papers) -Children’s details -Mental health status (where provided)	Aims	Qualitative methods	Reported data analysis
(Lesser et al., 1999) [40] USA	*N* = 15; ‘adolescents’ Parenting: 2 years postpartum Sample of ‘adolescents’ not defined in terms of age but quotations from young women age 17 (*n* = 1); age 18 (*n* = 5)	*N* = 11 Latina; *n* = 3 African-American; *n* = 1 Caucasian. *N* = 8 had one child; *n* = 4 were pregnant with their second child; *n* = 3 had two children. Reported symptoms of depression either during pregnancy or postpartum, measured by the Center for Epidemiologic Studies Depression Scale (*n* = 14) or reported a history of suicide attempt(s) within the past year (*n* = 1)	To provide a description of the affective component of depressed adolescents’ maternal role by eliciting young mothers’ perceptions of their maternal roles and their experiences of depression.	Ethnographic interviews, 2 h long conducted	Content analysis
(Clemmens, 2002) [41] USA	*N* = 20, age 16–18 Parenting: 1–11 months postpartum	9 African American; 8 Hispanic; 3 White All had one child Depressive symptoms	To address the phenomenon of depression from the perspective of adolescent mothers. Explore their memories of feeling depressed after the birth of their babies.	In depth face to face interviews, 15–45 min (20 mins average)	Descriptive phenomenological design (Colaizzi, 1978)
(Renker, 2002) [42] USA	*N* = 40; age 18–20 Pregnant or parenting Average age for conception for the current pregnancy was 18.	*N* = 17 African American; *N* = 18 White; *N* = 5 teens Hispanic, American Indian, or mixed racial background.	To explore adolescents’ experiences of abuse in the year before and during pregnancy.	Structured and focused interviews 30–90 min	Generalized qualitative approach blended content/thematic analysis. (Creswell, 1994)
(Kennedy, 2005) [43] USA	*N* = 10; age 16–20 Pregnant or parenting	*N* = 7 Mexican American; *n* = 3 African American.	To explore urban adolescent mothers’ experiences with community violence; witnessing parental violence, familial physical abuse, and partner violence; their methods of coping and resistance.	Individual, open-ended interviews (45–90 min)	Grounded Theory (Strauss & Corbin, 1990)
(Stevens, 2006) [44] USA	*N* = 18, age 15–21 Parenting – at least 3 months postpartum	*N* = 6 Black; *n* = 7 White; *n* = 3 Black/White/Indian; *n* = 1 Black/White/Mexican; Mexican/White *n* = 1 Age of children: 0–3 months *n* = 2; 3–6 months *n* = 3; 6–9 months *n* = 3; 9–12 months *n* = 1; 12–24 months *n* = 5; 2–4 years *n* = 6	To explore how adolescent women who are parenting describe what “being healthy” means to them and how they define their own health needs.	Ethnographic methods Including in-depth interviews and participant observation, photovoice	Participants’ views /meanings of health as described in interviews and narratives of photographs (ethnographic and photovoice)
(Shanok & Miller, 2007) [45] USA	*N* = 42; age 13–19 39 pregnant; 3 parenting	*N* = 18 Hispanic; *n* = 19 Black; *n* = 4 Black and Hispanic, *n* = 3 undisclosed Subsection with depressive symptoms	To explore the nature of the participants’ depression and the factors that helped them to feel better.	Mixed methods. Analysis of therapy sessions with participants	Inductive qualitative analysis LeCompte and Schensul (1999)
(Erdmans & Black, 2008) [46] USA	*N* = 27; average age 20, Parenting Average age 17 years old when they had their first child	12 White, 9 Puerto Rican, 5 African American, and 1 biracial (White and Puerto Rican).	To listen to victims of child abuse tell their life histories to better understand the trajectories linking child sexual abuse to adolescent motherhood.	Two face to face interviews, 1.5–2 h	Life story method (Bertaux & Kohli, 1984)
(Meadows-Oliver, 2009) [47] USA	*N* = 8; age 18–19 Pregnant (*n* = 2); parenting (*n* = 5)	*N* = 7 African-American, *n* = 1 Latina. *N* = 4 one child; *n* = 4 two children – ages between 7 months to 5 years. *N* = 2 pregnant at time of interview	To explore the lived experience of homeless adolescent mothers’ caring for their children while living in a shelter?	1:1 face to face interviews lasting 20–30 min	Phenomenological approach (Colaizzi, 1978)
(Kulkarni, 2009) [48] USA	*N* = 24, age 18–22 Parenting Age at first pregnancy 14–16 *n* = 19; 17–18 *n* = 5	African American *n* = 9; Asian *n* = 1; European American *n* = 4; Mexican/Mexican American *n* = 10 Number of children 1 *n* = 18; 2–3 *n* = 6	To explore the effects of IPV on their adolescent mothers’ important relationships.	Semi structured interviews 35 mins to 2 h with *n* = 24; second interviews with *n* = 15	‘Qualitative analysis’ (Miles and Huberman, 1994)
(Brown, Brady & Letherby, 2011) [49] UK	*N* = 9; age 16+ Parenting (1–5 years) Age range time of giving birth 13–18 years.	Children age 1–5 years	To explore a range of issues pertinent to young women’s lack of agency, disempowerment and experiences of power, control and domestic violence with reference to intimate and familial relationships.	In-depth, semi-structured interviews 1–2 h	Grounded theory “style” (Glaser & Strauss, 1967)
(Smith & Roberts, 2011) [50] UK	*N* = 13 under age of 25 at interview Parenting	*N* = 5 White British; *n* = 3 Mixed race; *n* = 2 Black British; *n* = 1 Indian; *n* = 1 Caribbean; *n* = 1 Black African	To explore the experience of being a young parent and some of the influences on their sexual and reproductive behaviours in young mothers from a variety of socioeconomic backgrounds.	Semi structured interviews	Thematic analysis (Braun and Clarke, 2006)
(Crawford et al., 2011) [51] USA	*N* = 24, age 16–19 at baseline Pregnant or parenting	Data not provided for the sub sample of those interviewed for qualitative research.	To follow a sample of young homeless females over a 3-year period as they moved from late adolescence into early adulthood informed by in-depth interviews with a subsample.	Semi structured interviews of 1 h	Thematic coding
(Boath et al., 2013) [52] UK	*N* = 15; age 17–19 Parenting - first time mothers with babies under the age of one. Age range at the time of giving birth 16 to 18 years (mean 16.9).	Children under age of one Identified by their health visitor as suffering from postpartum depression following clinical assessment.	To elicit the experiences of teenage mothers with postpartum depression and to further explore those factors associated with depression in younger mothers.	40 min – 1.5 h face-to-face semi-structured interviews	Thematic framework analysis (Ritchie & Spencer, 1994)
(Herrman, 2013) [53] USA	*N* = 26; age 14–18 *N* = 22 16–39 weeks pregnant; *n* = 4 parenting	*N* = 7 Hispanic; *N* = 10 African American; *N* = 6 White; *N* = 3 mixed origins Children age 1–3 months.	To provide a voice to young mothers about their thoughts and perceptions of TDV within the context of their relationships and experiences in pregnancy and parenting.	Semi structured focus groups	Qualitative coding of typologies (Rubin and Rubin, 2005)
(Aparicio et al., 2015) [54] USA	*N* = 6; age 19–22 Parenting, mothers in foster care Age 14 to 17 years at the time of their first pregnancy.	*n* = 5 African American; *n* = 1 Latina, born in the U.S. Three participants had one child, one participant had two children, and one participant had three children.	To explore the lived experience of motherhood among teen mothers in foster care with a history of maltreatment. To inform teenage pregnancy prevention and to elucidate practices to give teenage mothers in foster care and their children the very best start possible in cases where a birth occurs.	Three in-depth interviews for each participant 1–2 weeks apart	Interpretative Phenomenological Analysis (IPA)
(Kinser & Masho, 2015) [55] USA	*N* = 17; mean age: 17.5 +/− 1.3 years Pregnant	*N* = 17 African American	To evaluate pregnant, AA adolescents’ perceptions of depression and stressful experiences and assess feasibility and acceptability of adjunctive/ complementary, non-pharmacologic stress and depression management strategies for this underserved population.	Qualitative interviews using nontherapeutic focus groups	Content analysis with phenomenological overtones (Sandelowski, 2000)
(Leese, 2016) [56] UK	*N* = 12; age 16–19 Parenting	–	To capture young mothers’ journeys to understand the reality of individual and collective experiences within cultural context of support group (Flick 2009).	Ethnographic narrative interviews and participant and non-participant observation collected over year	Thematic analysis Gomm (2008)
(Fortier & Foster, 2017) [57] Canada	*N* = 10; age 21–25 Parenting Conception age 13–19	All identified as Anglophone and Caucasian	To understand better the experiences of young mothers with subsequent pregnancy and motherhood in Canada’s capital.	Semi structured depth phone interviews av. 60 mins	Qualitative content analysis (Elo and Kyngäs 2008)
(Bledsoe et al., 2017) [58] USA	*N* = 20; age 14–20 Pregnant	46.5% African American; 46.5% Latina. Pregnant: average gestational age 16.85 weeks (SD = 4.63) 6 prior depression diagnosis; 1 bipolar; 1 mood disorder,1 panic disorder	To address knowledge gaps regarding the experience of U.S. low-income, minority, depressed pregnant adolescent women’s perceptions of pregnancy, depression, and help-seeking.	In depth, semi structured interviews and questionnaire	Descriptive qualitative approach

The included papers reported on a total of 356 young women aged between 13 and 25 years old, who became pregnant under the age of 20. Two studies were focused only on pregnant women [55, 58], eleven studies involved young women who were parenting [40, 41, 44, 46, 48–50, 52, 54, 56, 57]. The remaining six studies included both pregnant and parenting young women [42, 43, 45, 47, 51, 53]. Young women’s ethnicities were reported in varying ways, but the majority identified as African American, Latino or Hispanic, or White. Young women in three studies identified as homeless – living in sheltered accommodation – [47, 51, 54] and another four studies reported that all the young women in their studies had low socioeconomic status/low income [43, 55, 56, 58]. Participants in five of the studies were sampled for specific symptoms of – or diagnosis of – depression [40, 41, 45, 49, 58]. In seven studies, all the included young women were reported to have experience of abuse or violence [42, 43, 46, 48, 49, 53, 54] (see Table 2 for further details).

Quality appraisal found that 15 out of the 19 papers were of reasonable quality. Common weaknesses of the remaining studies included: not outlining why particular methods had been chosen [42, 51]; not explaining how participants had been recruited or any issues in doing so [42, 50, 51]; not detailing how sample sizes were decided or when saturation was reached [50, 51]; failing to discuss the researcher’s position or potential influence on the study [42, 45, 50, 51]; and a lack of sufficient rigour in the presentation of the findings [42, 45, 50, 51].

### Outcome of relating studies: how studies relate to each other

As the second order constructs were reviewed, we began to see that the studies did not refute each other, but spoke to common constructs around mental health and wellbeing, so the synthesis was not refutational (studies did not provide opposing accounts) but reciprocal, in that studies developed similar concepts that could be fed into one another. Indeed, the studies provided comparable accounts of perceptions of mental health and wellbeing during and after pregnancy. Despite coming from different countries and speaking to young women at different time points, there were no clear subgroups of analysis as the primary studies emphasised a common base of factors relating to women’s mental health and wellbeing.

### Outcome of translation and synthesis process

**Fig. 2:**
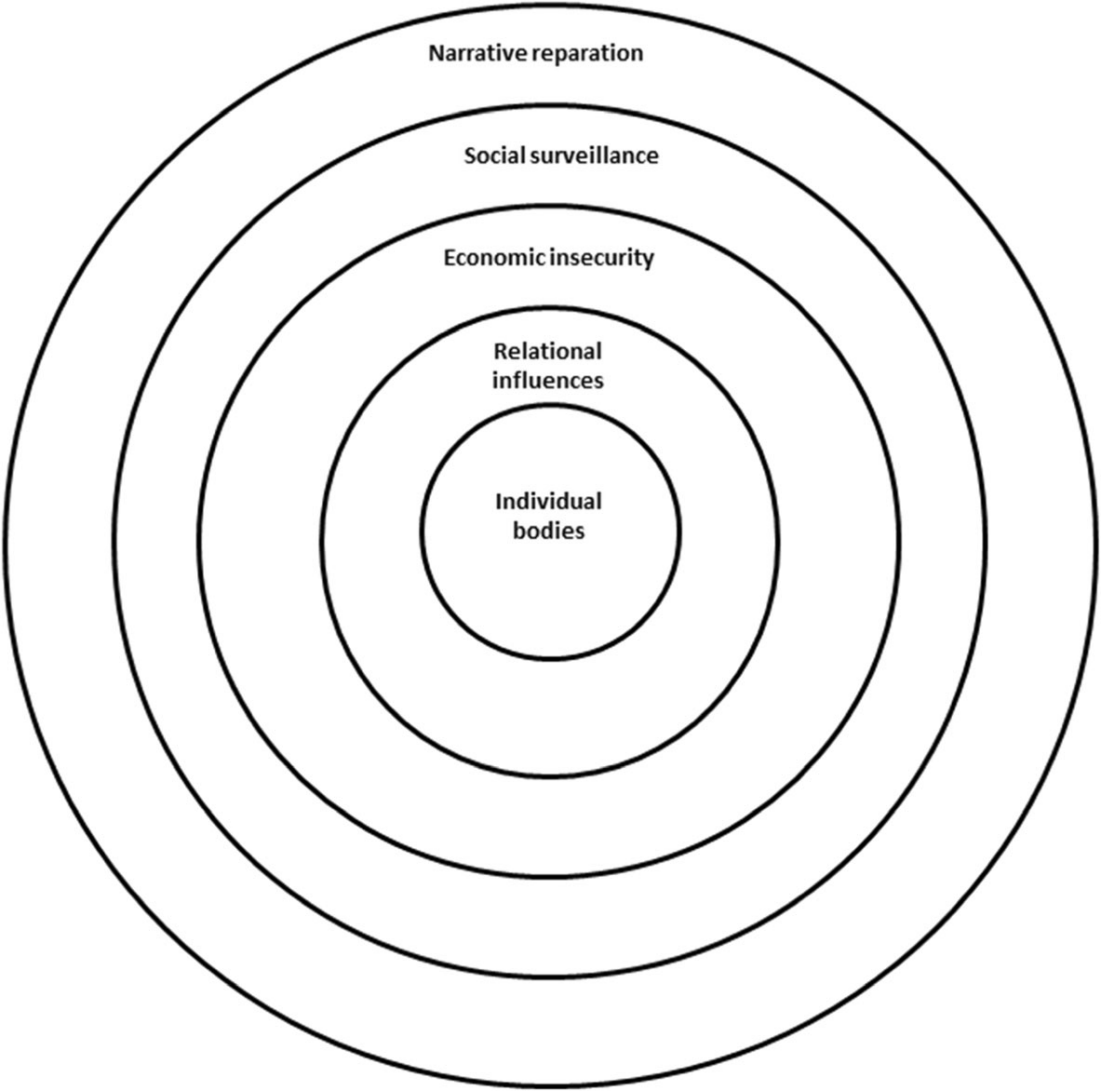
Line of argument synthesis: ‘No straight lines'

The synthesis of nineteen papers led to the development of seven themes (and related sub-themes) (see Table 3), which create a line of argument to help understand young women’s experiences of mental health and wellbeing during and after pregnancy. The line of argument in this synthesis is not so much a line but mapped as a series of circles that help to understand and make sense of young women’s mental health and wellbeing experiences (see Fig. 2: Line of argument synthesis: ‘No straight lines’).

**Table 3:** Translation of second order constructs and construction of line of argument synthesis (themes)

Line of argument	Synthesis 3rd order interpretations (or themes)	List of 15 translated 2nd order constructs (sub-themes)	Definition (translation) of the 2nd order construct	Papers that include the 2nd order constructs
Individual bodies	Embodied trauma: Deep imprints	‘Living with violence’ [43]	Interpersonal violence in YW’s lives impacts physically and mentally. Violence is tolerated due to low self-esteem and as a familiar pattern in lives.	[40, 43, 49, 51, 53, 57]
		‘Crying out for help’ [42]: Traumatic after effects of childhood	Abusive, conflictual, violent relationships from childhood contribute to later depression as trauma which has been suppressed resurfaces. Suicidal attempts and self-destructive behaviour linked to childhood abuse; women propelled into intimate relationships early. Abandonment and loss also involved in complex traumatic histories.	[40, 42, 43, 46, 48, 54, 58]
	Stress and overwhelm: Weight on shoulders	‘Carrying all the stress’ [54]: emotionally and physically draining	Stress in pregnancy; stress of being pulled between adolescent and mothering roles and having to adapt to responsibility is emotionally and physically draining; stress of children acting out; stress of living circumstances.	[41, 47, 51, 52, 54, 55, 58]
		Stress: ‘increasing the risk’ [53]	Stress of pregnancy and motherhood raises risk of violence, depression and suicidal attempts and leads to fatigue and overwhelm.	[43, 44, 47, 51, 53]
	Not just hormones: Depression darkness	Depression impact on the ‘transition into motherhood’ [56]	Post-natal depression is present, difficult and, for some mothers, causes difficulty parenting	[40, 51, 56, 57]
		Difficulty of identifying and ‘explaining the unexplainable’ [41] of depression	Depression figured as blinding and feels like explaining something unexplainable. YW interpret it as stress or relate it to life events, relationships and circumstances.	[41, 45, 48, 52, 55, 58]
Relational influences	Held together: support, conflict and isolation	‘Circle of support’ [54]: sustaining and protecting	Family or wider circles of support can provide validation and increase esteem and lessen stress of pregnancy. Adult relationships can be enabling, provide sense of new pathways and help YW seek help for m/health issues.	[41, 45, 48, 52–54, 58]
		‘Interpersonal disputes and conflict’ [58]	Conflict in relationships with partners, family members or other people, and emotional violence or controlling relationships are also trigger for stress and depression as well as for homelessness.	[40, 44, 49, 51, 58]
		‘Left behind’ [41]: social isolation	Despite some sources of support, YW feel social isolation from a sense of abandonment by partners (in particular); friends or family due to being a young mother; or because YW have chosen to stay away from bad influences or violent relationships. Result of isolation or sense of abandonment or exclusion may be a sense of depression, loneliness and suicidal thoughts.	[40–42, 45, 48, 52, 55, 56, 58]
Socio-economic insecurity	Unstable foundations: Ground beneath feet	Impact of ‘housing instability’ [58] on mental health and wellbeing	Housing instability and/or homelessness and/or tumultuous living conditions impact development and transition to motherhood. Living circumstances lead to or seen as factor in depression.	[42–45, 47, 49–51, 54, 56, 58]
		Impact of ‘socioeconomic stress’ [44] on mental health and wellbeing	Economic stressors mean YW do not have what need to live healthy lives; poverty primary factor in depression and increased stress, leads to feelings of despair.	[44, 50, 54, 58]
Social surveillance	Surveilled and judged: Head down	Impact of ‘stigma and perceptions of being judged’ [52] on help seeking for mental health	Mental health is seen as a stigmatised or judged issue, which, along with the stigma and judgement associated with being a teenage mum, prevents help seeking, and is hidden from HCPS, as YW try to present themselves as good mothers.	[52, 56, 58]
		Impact of ‘stigma and perceptions of being judged’ [52] on emotional life world	Judgement from the public or community about being a young, single mother adds to stress, and contributes to depression and to social exclusion. Impact of stigma and perceptions of being judged has a negative impact on emotional wellbeing.	[41, 44, 45, 49, 52, 56]
Narrative reparation	Empowerment and resilience: Breaking cycles and managing impressions	‘Light in the darkness’ [54]: reparation and empowerment of motherhood	Repairing childhood wounds, positive changes in mental health and wellbeing in becoming a mother including increased motivation, feelings of love for children, opportunity to return to education, positive maternal behaviours, breaking cycles. Pregnancy provides a point to move on from harmful behaviours.	[40–44, 46, 51, 53, 54, 56, 57]
		‘Impression management’ [56]	Young women present stories, in a positive light in order to avoid the ‘stigma’ attached to teenage motherhood and mental illness.	[40, 44, 54, 56]

### Individual bodies

The first circle perceived to impact young women’s mental health and wellbeing related to individual women’s bodies and forms the centre of the line of argument where most emphasis fell across the studies. It is underlined by three themes: Embodied trauma, Stress and overwhelm, Not just hormones.

#### Embodied trauma: deep imprints

‘Living with violence’ [43], (p. 1940): The effect of abuse and violence in young women’s lives and the impact on their mental health was a recurrent concept across the studies. Young women were exposed to violence before, during and after pregnancy [40, 43, 51, 57]. Some women came to tolerate it due to low self-esteem as one participant explained: ‘I used to think that I deserved it [ …] I was so down on myself at the time’ [49], (p. 369) and because it was an all too familiar pattern in their lives [53]. This violence had a debilitating impact on health and wellbeing [43, 49].

‘Crying out for help’: For some young women, violence and abuse had been a part of their lives from a young age, forming a deep imprint and affecting their mental health. As women became mothers themselves, as one study reported, they were ‘crying out for help’ [42], (p. 111) as trauma which had been ‘blocked’ or ‘stuffed [ …] away’ resurfaced [46], (p. 84-5). In some cases, studies reported that this led to suicidal attempts [42], depression [40, 48] and self-destructive behaviour [48]. Abandonment and loss were also involved in complex traumatic histories and in young women’s feelings of despair [54, 58].

#### Stress and overwhelm: Weight on shoulders

‘Carrying all the stress’ [54], (p. 51): Stress was a theme in several studies [41, 47, 52, 54, 55, 58]. As Meadows-Oliver [47] identified, this stress was ‘emotionally and physically draining’ (p. 464). Stress started in pregnancy for some young women [51, 55] and continued as the ‘sudden realisation’ [41], (p. 557) of motherhood and the strain of being pulled between – or having to balance – adolescent and mothering roles became a reality [41, 43, 53, 58]. Young women had to adapt to new levels of responsibility as one young woman articulated:

Nobody, gives me a hand with her. Where can I go for support, because as soon as I came out of hospital I just come home and I thought, what a life and what have I got at this age …[52], (p. 361).

‘Increasing the risk’ [53], (p. 465): Stress was reported to relate to young women’s broader living circumstances [47]. The stresses young women faced in their living situations were reported to lead to an increase in violence in their relationships [43, 51, 53] and contributed to depression [44], fatigue and overwhelm [47] and suicide attempts [44]. One young woman described how the stress of her earlier life was physically experienced as a burden carried ‘uphill’ on her shoulders [44], (p. 35).

#### Not just hormones: depression darkness

‘Transition into motherhood’ [56], (p. 521): Three studies discussed how the transition to motherhood was made more difficult by mental health issues including depression [40, 51, 56]. Young women felt that they would not be understood and would be judged to be a ‘bad mum’ [52] if they had depression and so, as one participant explained, they kept things ‘bottled up’ (p. 356).

‘Explaining the unexplainable’ [41], (p. 558): Some young women did not know the meaning of depression as exemplified by one young woman:

Nobody has ever told me what it is really [postpartum depression] … I just sit here sometimes and I am crying for no reason, but I could have detected it earlier if someone had explained to me what your first symptoms were, but nobody told me [52], (p. 361).

As one young woman put it, the feelings were more than ‘just’ pregnancy hormones [58], (p. 254). Young women’s language around their symptoms was reported across the included studies to be different to that of screening criteria for identifying depression [41, 58]. Young women expressed their feelings as overwhelm or stress [58] or they focused on their life circumstances as the main issues, rather than the depressive symptoms themselves [45, 58].

### Relational influences

The second circle relates to the people that immediately surround young women during and after pregnancy. This circle is underpinned by one theme – Held together: support, conflict and isolation – that captures the complex dynamics of relationships and their impact on young women’s mental health and wellbeing.

#### Held together: support, conflict and isolation

‘Circle of support’ [54], (p. 49): Circles of family or social support around young women could be powerful in helping young women’s sense of wellbeing [41, 52]. These relationships could lessen the stress young women were facing in pregnancy and motherhood [53]. Family and community members provided positive validation, which increased young women’s sense of feeling good [45, 48] and enabled them to develop new and positive adult relationships [54]. This could lead to women seeking professional help for mental health problems if needed [58]. The presence of babies’ fathers was highlighted as potentially most important, as the critical times when women needed support (for example in the middle of the night) were often those times when wider family members were not around [41, 52].

‘Interpersonal disputes and conflict’ [58], (p. 252): As much as relationships could be a positive influence on young women’s wellbeing, relationships could be equally cause conflict and more stress during and after pregnancy [40]. Negative relationships with others added to young women’s levels of stress [44] and were reported to be a key factor in women’s experiences of depression [58]. Emotional belittling by others impacted young women’s sense of confidence and self-esteem [49]. In some cases, the influence of significant family members could prevent – rather than enable – service use [49, 58].

‘Left behind’ [41], (p. 559): Young women’s role as a mother sometimes cut them off from peers [41, 55] and it was worsened when they were rejected by family members [45], or left unsupported by the babies’ fathers [52, 55]. From pregnancy and into parenting, some women described feeling abandoned [40, 41] and depression started there:

The friends that I used to have, I don’t have. So, that is another reason why I am depressed because they say, ‘Oh!’ I can’t go out with them or hang out with them no more so they are not going to hang with me or call me anymore. I honestly have no friends. And you know, it’s a lot of things because people look at me now differently because they see me carrying a baby [41], (p. 559).

Social isolation both reflected and impacted upon depression [40, 48, 55, 58] and could end in a sense of all-encompassing loneliness [56]. Sometimes, young women’s drive to change things in their lives [40] and leave harmful or violent relationships, meant they were further cut off, if they took that step [42] with the ‘circle of support’ [54] feeling ever diminishing.

### Socio-economic insecurity

The third circle represents the broader socio-economic contexts of young women’s lives. Young women felt that wellbeing and good mental health were hard to achieve without somewhere stable to live and when children’s basic needs could not be met. Mental health and wellbeing came from a sense of security in the ground beneath their feet and formed the theme in this circle.

#### Unstable foundations: ground beneath feet

‘Housing instability’ [58], (p. 254): Young women recognised the basic need for housing needs to be fulfilled to ensure health and wellbeing [44]. For young women without stable living circumstances, the ability to take the next step in their development and adjust to their new role as a mother was impeded [42]. For young women in this position of housing instability, trying to find secure housing came to dominate life [47, 56]. Conflict and violence in relationships meant that homelessness was a real possibility should women try to leave [43, 49, 51] and young women’s sense of feeling trapped was reported to lead to depression and anxiety [43, 45]. Housing was a cause of stress both during and after pregnancy as young women worried about the environment into which they were bringing their children [50]. As one young woman explained, raising your child in a shelter, ‘takes a lot of energy. It’s stressful, hard. It’s a big toll.’ [47], (p. 462). Housing instability and continual uprooting also created a ‘practical barrier’ to young women’s ability to seek help and access services for mental health problems [58], (p. 254).

‘Socioeconomic stress’ [44], (p. 37): ‘Multiple and compounding economic stressors’ [58] meant that young women did not have what they felt they needed to live what they considered to be ‘healthy’ lives [44], (p. 28). Not having enough money to make ends meet led to sense of what Aparicio et al. [54] termed ‘darkness and despair’ (p. 47). Young women also perceived socioeconomic stressors including unemployment, limited resources and poverty-related stress as the significant problem impacting their mental wellbeing [50, 58]. The fulfilment of ‘basic needs’ was analysed to be vital before any sense of wellbeing could be felt [44], [54], [58], (p. 255). It was also the tipping point from which new cycles of poor mental health might start, as one young woman explained:

I didn’t want my child to grow up like how I grew up, like from place to place and people neglecting them or, you know, and things like that. I just wanted him to grow with a normal childhood like a child should [54], (p. 48).

### Social surveillance

The fourth circle represents the impact on young women’s mental health and wellbeing during and after pregnancy of perceived social judgement and stigmatisation. For some young women, additional stigmatisation related to mental illness was also reported. This is explored in the theme: Surveilled and judged: head down.

#### Surveilled and judged: head down

‘Stigma and perceptions of being judged’ on help seeking [52]: Young women perceived that mental health problems should be concealed, as they struggled with perceived judgement and stigma from those around them [56, 58]. Those who felt under scrutiny from professionals regarding their parenting skills did not want to admit to more issues which may affect how they were viewed, as one woman explained:

I don’t know what the health visitors are like, or if they are going to say things or twist things round to say that I can’t cope. The thing is, with health visitors they’re scared of them going to social services, which is the main concern, that’s why people don’t speak out about postnatal depression, they keep things bottled up [52], (p. 356).

These young women felt that they had to represent themselves as ‘good mother [s]’ and believed that mental health problems contradicted the narratives they were expected to conform to [56]. As Leese analyses, they felt they should ‘act out’ what they perceived to be the right version of motherhood [56].

‘Stigma and perceptions of being judged’ on emotional life world [52], (p. 355): Perceived judgement and stigma affected young women’s wellbeing. Young women reported feeling publically scrutinised [41] and judged for being a teenage mother [56]. As one young woman put it, ‘See, I don’t even get the courtesy of the hand over the mouth; I get people in the shopping centre staring at me’ [52], (p.356). These judgements added to young women’s stress and made them feel defensive [44] or increased feelings of anger and sadness [45]. It also affected their sense of competency and self-esteem as mothers [52]. In some cases, women reported feeling that they could not leave violent relationships as they did not want these judgements to be compounded by the status of being both single and a young mother [49].

### Narrative reparation

The final circle describes how young women’s narratives are presented as stories of overcoming, resilience as a form of wellbeing, and linear ideas of journeying from darkness into light. However, these narratives do not always take the form of straight lines. This circle is explored in the theme: Empowerment and resilience: breaking cycles and managing impressions.

#### Empowerment and resilience: breaking cycles and managing impressions

‘Light in the darkness’ [54], (p. 49): For some young women, pregnancy and motherhood was articulated as a time of reparation that positively influenced their wellbeing. In some cases, this reparation was linked to working through childhood wounds, where young women realised they were not to blame for what happened to them as children [46]. Being a mother acted as a source of motivation [57] and inspired a desire to do things differently [54, 56]. Young women had to draw on previously untapped strength [40] and, for some, it helped improve self-esteem [56]. As Kennedy [43] analysed, resilience was related to ‘intraindividual capabilities’ (p. 1503). Some young women possessed an outlook that enabled them to deal with adversity:

So I guess instead of saying teen moms are smarter, I should say they are stronger. .. It’s like you are in a race with someone who’s not pregnant or doesn’t have kids, in the same race with them, running the same amount of time, but theirs is maybe 5 miles, and mine is maybe 10 … that’s the way I see it. I′m running a lot more, but we’re still in the same spot [40], (p. 141).

Some women perceived that having a child gave them a more future-orientated outlook [51], including the desire to return to education [40, 41, 54] and helping by ‘breaking the cycle’ of violence [53], (p. 468). In relation to physical wellbeing, pregnancy and motherhood was reported to offer an opportunity to turn away from behaviours that ‘harm your body’ [44], (p. 32) including drug use [40, 42, 51] alcohol consumption [57] or smoking [44, 57]. Study authors used a range of metaphors to convey the idea of a positive, upward journey for young women’s wellbeing after becoming a mother including: the sun breaking 'through the storm clouds' [41], (p. 561), ‘glimpses of light in the darkness’ [54], (p. 49) and ‘turning the corner’ [56], (p. 526).

*‘*Impression management’ [56], (p. 525): Young women’s stories were not all about overcoming, as their lives were also reported to be lived ‘on the edge’ [42], (p. 111). As Leese argued, because young women were trying to demonstrate that they were good mothers, they were focused on ‘impression management’ [56] [59]. They therefore created and articulated narratives that conformed to what they felt were acceptable or desirable. Indeed, studies reported that young women wanted to show they were ‘the same’ [40], (p. 141) or no different [44] from older mothers. As one young woman articulated, in the study by Aparicio et al. [54], her main aim was to ‘prove’ herself to counter the ‘statistics’ of teenage motherhood (p. 45). This was also specifically an issue in relation to mental health, which it was felt would be viewed as related to poor parenting [56].

## Discussion

### Summary of findings

The aim of this meta-ethnography was to synthesise international evidence from qualitative studies focused on young women’s perceptions of their mental health and wellbeing experiences during and after pregnancy. Whilst mental health is often constructed in individual terms as an issue of the mind or brain [60], the first circle in this meta-ethnography suggests that young pregnant and postnatal women’s distress (often relating to traumatic life events) is experienced physically. As has been reported in previous research, this was often related to previous experiences of violence and physical abuse, as well as emotional abuse [18]. Women’s experiences of depression as dark and inexpressible related not only to the sense that they could not describe their feelings, but also that they should not disclose their feelings, fearing judgement in relation to their ability to parent. This inexpressibility also related to the way in which young women did not recognise those feelings as problems of mental health but of living through difficult life circumstances.

The second circle through which young women’s mental health and wellbeing during and after pregnancy was experienced was via their relationships with others. Social connection and support could help young women’s wellbeing during and after pregnancy, underlining findings of previous research [61]. Conversely, young women felt that a lack of support created isolation and stress. Indeed, a lack of social support has been linked with increased depression [62, 63]. However, family conflict, violence and interpersonal disputes were often attached to those providing support. Previous research suggests that family can act both as a support and a barrier to women accessing help for postnatal depression [58] and that it is who the support is coming from that may be important [64], with poor levels of support from the baby’s father related to depression postnatally [65, 66].

The second circle was the connecting layer between young women’s individual bodies and their broader social world and whether they had adequate resources, housing and access to services, in the third circle representing their sense of socio-economic (in)security. This meta-ethnography adds further evidence to suggest mental health problems are experienced within and influenced by the external conditions of women’s lives and relate to economic stressors. The role of housing instability and financial and food insecurity in young women’s lives figured highly confirming other research [67, 68]. Indeed, there was little variation in the narratives of young women during and after pregnancy suggesting that the socioeconomic complexities underwriting young women’s lives remained dominant throughout. This synthesis suggests that young women recognise stress as profoundly impactful, and want to avoid it, but find that it is exacerbated by their socioeconomic situation. This emphasises the need for interventions that target social factors to impact long term mental health outcomes [69].

The fourth social surveillance circle of this synthesis adds further evidence to suggest the links between the stigma and wellbeing [70]. Given that surveillance frames teenage pregnancy in certain countries – the fact that rates are targeted to be reduced and it is set up as a problem from the outset – arguably worsens young women’s mental health as the sense of perceived judgement affects their sense of emotional wellbeing [71, 72].

Rather than there being simple stories of overcoming or triumphant resilience in the form of individuals directing their lives into straight, upward lines, young women’s stories were much better characterised as layered with complexity. The multi-layers of women’s perceptions of mental health and wellbeing during and after teenage pregnancy are comparable to Bronfenbrenner’s ecological systems theory of development [73]. This theoretical approach centralises the influence of different environmental systems on development including that of the microsystem of immediate family and community influences, and the macrosystem of culture, and supports the contextual approach that this meta-ethnography provides.

The outer circle of the synthesis considers the role that narrative itself plays in how young women come to understand and position their mental health and wellbeing experiences during and after pregnancy. Fearing judgement and stigmatisation for young motherhood and mental health problems, young women in these studies appeared to want to provide narratives to fit perceived models of success or resilience. Critical work around ‘illness narratives’ in medical humanities scholarship has explored how narratives may be used as a tool to open up a window to experience and re-establish notions of self [74]. However, as cultural studies scholar Lisa Blackman puts it, culturally preferred narratives are those of triumph and overcoming: ‘the victim-to-victor narrative is one where mental distress becomes both a site of self-knowledge and identity work’ [75], (p. 8). This emphasis on having to tell a ‘good’ story [76], (p. 60) may also be influenced by the way in which narratives are constructed by the authors of the papers included in this synthesis, who mostly (with the exception of Leese [56]) appear to take a straightforward approach to narrative as that window onto experience. Furthermore, metaphors chosen to illustrate the journeys of young women appeared to follow a darkness into the light approach [41, 54, 56]. A more critically orientated reflection on the constraints and models of narrative available, as Anastas (2017) [77] has provided about teenage motherhood itself, is particularly required in the domain of mental health. Healthcare professionals and policy makers may benefit from reflecting on the impact of particular scripts and narratives on young women’s sense of health and wellbeing.

### Strengths, limitations and reflexivity

It is perhaps not surprising that in a search focused on young mother’s mental health and wellbeing and their associated terms that the majority of studies retrieved focused on young women’s struggles or problems. Six studies aimed to explore depression [40, 41, 45, 52, 55, 58] seven set out to explore issues of abuse, violence or unequal power relations [42, 43, 46, 48, 49, 53, 54] and three studies looked at socio-economic issues in young women’s lives including homelessness [47, 50, 51]. The young women recruited in the original studies and the aims and objectives of the researchers involved needs to be reflected upon. As this review was limited to peer-reviewed papers published in academic journals and searched via health-related databases it was arguably limited to areas of enquiry focused in domains of health and social care. Indeed, the majority of authors of these articles are from the fields of nursing (*n* = 8), social work (*n* = 5) or psychology (*n* = 3) and are therefore directed to the implications for public health and practice, with research funded and constructed in intervention-focused frameworks within countries where teenage pregnancy has been identified as a public health issue. Indeed, the included studies were predominantly from the USA, with only a few from the UK and one from Canada. Papers from other OECD countries were located in the search but did not to meet the criteria for this particular synthesis, which focused on research with a primary aim or results that clearly foregrounded mental health or wellbeing. For example, papers from an Australian context echoed the social problem focus for teenage pregnancy [78, 79]. Whilst the findings of this synthesis may resonate with contexts and countries where teenage pregnancy remains stigmatised, for other countries adolescent pregnancy is differently constructed and lacks the ‘crisis’ features apparent in the American and British contexts [80], (p. 147). A more open-minded approach to teenage sexuality in other countries may mean that stigma and ‘societal disapproval’ is reduced [81] and the distinct mental health and wellbeing experiences of young mothers are less frequently problematized or explored, arguably limiting the generalisability of these findings to some other OECD countries.

Indeed, the focus on mental health means that the papers included in this synthesis predominantly call attention to the challenges and difficulties of young mothers. There will be other young women who fall outside of the scope of these papers and are not recruited because studies are problem-focused and young women do not experience the issues being explored. Only one included paper took a positive approach to understanding what health and wellbeing meant for young women [44], although other papers did reflect on some enhancing aspects of motherhood for young women in general [40, 43, 49, 54, 56]. It is important to reiterate that not all stories of young motherhood involve mental health problems, hardship or psychological distress. Indeed, for some young women having a baby is a way of finding love; helping them to overcome and restore their lives [82].

The process of the selection and synthesis in a meta-ethnography is interpretative and therefore other teams of researchers may have come to a different conceptual framework. Whilst other reviewers were all involved in the interpretative process and synthesis findings, the initial detailed translations were conducted by one author and given the detailed level of extraction and translation this may have somewhat guided the initial synthesis process.

## Conclusions

This is the first review of young mothers’ perceptions of their mental health and wellbeing using a meta-ethnographic approach and following eMERGe reporting guidelines [28]. It provides new insights into how problematizing teenage motherhood stigmatizes it and how stigma negatively impacts young women’s wellbeing. It additionally critically questions individualized resilience-based or overcoming narrative frameworks and identifies the need for broad based model of health and wellbeing rooted in relational, social and economic contexts. Whilst the review synthesises papers from three countries, evidence suggests that young women from other countries where teenage pregnancy is stigmatised or distinctly problematized may have similar experiences. Indeed, this novel synthesis suggests that how research is framed requires careful consideration, given the double stigmatisation around young motherhood and mental health in some countries. Indeed, the assumptions and societal narratives that frame research into teenage motherhood are powerful [83, 84], and suggest the need for reflection from researchers and professionals working with young women in terms of understanding how they may unintentionally reinforce power dynamics or add to stigma. Given the detrimental effects of stigma, this review suggests a holistic and non-judgemental approach to supporting young women may be beneficial; one which takes a strengths or asset based view of young mothers’ potential. New attempts to ‘re-assemble’ stories of teenage pregnancy [85] provide some alternative perspectives to those of health or social care, where the emphasis is often aimed at fixing or ameliorating problems in a social crisis model of teenage pregnancy. Research that does not begin with a deficit view and seeks to specifically explore the meanings of mental health and wellbeing for young mothers without starting from a problem or crisis based framework is needed. Innovative research methodologies that use alternative embodied methodologies [86, 87] or auto-ethnographic methods that de-stabilise the power hierarchy of researcher and participant (as authors are both researchers and former ‘teenage’ mothers [88, 89]) may be useful for researchers in undoing some of the more limited models of conceptualising teenage pregnancy in countries where teenage pregnancy remains a stigmatized issue. Indeed, despite coming from three different countries, the evidence in this meta-ethnography points to common issues for these young mothers and the need for healthcare professionals and policy makers alike to think more holistically about mental health and wellbeing, which is powerfully presented as enmeshed with the social and economic structures of young women’s lives.

## Data Availability

The datasets analysed during the current study are available from the corresponding author on reasonable request.

## Abbreviations

CASP: Critical Appraisal Skills Programme
MeSH: Medical subject headings
OECD: Organisation for Economic Co-operation and Development
PRISMA: Preferred Reporting Items for Systematic Reviews and Meta-Analyses
